# Impact of dietary supplementation of *Bacillus subtilis* on the metabolic profiles and microbial ecology of weanling pigs experimentally infected with a pathogenic *Escherichia coli*

**DOI:** 10.1186/s40104-025-01313-7

**Published:** 2025-12-06

**Authors:** Kwangwook Kim, Sangwoo Park, Cynthia Jinno, Peng Ji, Yanhong Liu

**Affiliations:** 1 Department of Animal Science, Michigan State University, East Lansing, MI 48824 USA; 2 Department of Animal Science, University of California, Davis, CA 95616 USA; 3 Division of Animal Bioscience and Integrated Biotechnology, Gyeongsang National University, Jinju, South Korea; 4 Department of Hematology and Hematopoietic Cell Transplantation, Beckman Research Institute of City of Hope, Duarte, CA 91010 USA; 5 Department of Nutrition, University of California, Davis, CA 95616 USA

**Keywords:** *Bacillus subtilis*, Colon microbiota, Enterotoxigenic *Escherichia coli* F18, Metabolomics, Weaned pigs

## Abstract

**Background:**

Our previous study demonstrated that dietary supplementation of *Bacillus subtilis* enhanced growth performance and intestinal integrity in weaned pigs challenged with enterotoxigenic *Escherichia coli* (ETEC). Therefore, this study aimed to explore the impact of *Bacillus subtilis* on gut health and its role in modulating host–microbe interactions in post‐weaning pigs.

**Results:**

ETEC infection disrupted key metabolic pathways in distal colon, including glutathione, beta-alanine, and pyrimidine metabolism, indicating increased oxidative stress, impaired nucleotide balance, and amino acid catabolic stress. *Bacillus subtilis* supplementation induced distinct metabolomic and microbiome profiles in colon digesta of weaned pigs challenged with ETEC. *Bacillus subtilis*-treated pigs under ETEC challenge exhibited significant enrichment in amino acid- and energy-related pathways such as arginine biosynthesis, phenylalanine metabolism, pantothenate and CoA biosynthesis. ETEC infection induced microbial dysbiosis in the distal colon, resulting in decrease (*P* < 0.05) in abundance of Streptococcaceae and Enterobacteriaceae compared to healthy controls. *Bacillus subtilis* supplementation mitigated the ETEC-induced disruptions by increasing the relative abundance of beneficial bacterial families, including Lachnospiraceae and Bacteroidaceae.

**Conclusion:**

Supplementation of *Bacillus subtilis* improves intestinal health and resilience against ETEC challenge by mitigating infection-induced metabolic disruptions and gut dysbiosis in weaned pigs.

**Supplementary Information:**

The online version contains supplementary material available at 10.1186/s40104-025-01313-7.

## Background

Enterotoxigenic *Escherichia coli* (ETEC) is a primary cause of post‐weaning diarrhea in piglets, posing a serious threat to swine health and production [1, 2]. ETEC infection impairs gut epithelial integrity, leading to villus atrophy, increased gut permeability, and subsequent reductions in weight gain and feed efficiency [3, 4]. In addition to these overt clinical signs, ETEC infection induces complex metabolic disturbances and disrupts the balance of the gut microbial community in weanling pigs [5, 6]. Such dysbiosis may further exacerbate nutrient malabsorption and inflammation, underscoring the intricate interplay between host physiology and microbial ecology [7].

Addressing these challenges, supplementation with *Bacillus subtilis* has been shown to mitigate the detrimental effects associated with ETEC infection [8–10]. In our previous study, pigs receiving *Bacillus subtilis* under ETEC challenge exhibited enhanced growth performance and improved gut barrier function, as evidenced by higher average daily gains and reduced intestinal permeability [11]. This direct-fed microbial intervention modulated host intestinal physiology by upregulating genes that encode tight-junction proteins and mucus while concurrently downregulating pro-inflammatory cytokine-encoding genes in intestinal mucosa. Nevertheless, the mechanisms by which *Bacillus subtilis* alleviates ETEC-induced intestinal dysfunction remain incompletely understood. Emerging advances in metabolomic and microbiome profiling provide valuable tools for unveiling host-pathogen interactions. Metabolome captures global changes in small-molecule metabolites [12, 13], while microbial ecology analyses reveal shifts in gut taxonomic composition [3, 14]. The integration of these advanced approaches provides powerful insights into how microbial alterations influence host metabolic status and overall intestinal health [15, 16]. To bridge existing knowledge gaps, this follow-up study employs integrated metabolome and microbiome to systematically characterize shifts in the intestinal metabolic profile and microbial community composition during ETEC challenge. By linking these molecular and microbial changes to key physiological outcomes, we aim to identify the specific metabolic and microbial pathways that mediate the protective effects of *Bacillus subtilis*. This comprehensive approach not only reinforces the potential of *Bacillus subtilis* as a functional alternative to antibiotics but also advances our understanding of the complex host–microbe interactions in post‐weaning pigs.

## Materials and methods

### Animals, housing, experimental design, and diet

The protocol for this experiment has been reviewed and approved by the Institutional Animal Care and Use Committee at the University of California, Davis. A total of 48 weanling pigs (initial BW: 6.73 ± 0.77 kg; 21–24 days of age) with an equal number of gilts and barrows were stratified by sex and randomly assigned to one of four treatments in a randomized complete block design, with body weight and litter as the blocks and pig as the experimental unit (*n* = 12/treatment). Pigs were individually housed (pen size: 0.61 m × 1.22 m) in an environmentally controlled unit for 18 d including 7 d before and 11 d after the first ETEC challenge (d 0). The detailed experimental procedures were published in Kim et al. [11].

The four treatments included: 1) Negative control (NC): control diet, without ETEC challenge; 2) Positive control (PC): control diet, with ETEC challenge; 3) Single dose of *Bacillus subtilis* (SD): control diet plus 1.28 × 10^9^ CFU/kg *Bacillus subtilis*, with ETEC challenge; 4) Double dose of *Bacillus subtilis* (DD): control diet plus 2.56 × 10^9^ *Bacillus subtilis*, with ETEC challenge. Spray-dried plasma, antibiotics, and high level of zinc oxide exceeding recommendation and normal practice were not included in the diets. The experimental diets were fed to pigs throughout the experiment and diet formulation was reported in Kim et al. [11].

After 7-d adaptation, all pigs were orally inoculated with 3 mL of ETEC F18 for 3 consecutive days from d 0 post-inoculation (PI). The ETEC F18 was originally isolated from a field disease outbreak by the University of Illinois Veterinary Diagnostic Lab (isolate number: U.IL-VDL # 05-27242). The ETEC F18 expressed heat-labile toxin, heat-stable toxin b, and Shiga-like toxin 2. The inoculums were prepared by the Western Institute for Food Safety and Security at the University of California, Davis, and were provided at 10^10^ CFU/3 mL dose in phosphate buffer saline. This dose caused mild diarrhea in the current study, which is consistent with our previously published research [17, 18]. Growth performance, blood profiles, immune responses, and gastrointestinal markers of development were published in Kim et al. [11].

### Sample collections

Twenty-four pigs (6 pigs per treatment) were selected randomly and euthanized on d 5 PI near the peak of ETEC infection. The remaining pigs were euthanized on d 11 PI. The selection of necropsy time was based on the results of clinical observations and immune response parameters that were reported in previously published research using the same ETEC strain and inoculation dose [17, 18]. Before euthanasia, pigs were anesthetized with a 0.5-mL mixture of 100 mg Telazol, 50 mg ketamine, and 50 mg xylazine (2:1:1) by intramuscularly injecting into the ham of the hind leg. After anesthesia, an intracardiac injection with an overdose of sodium pentobarbital (Vortech Pharmaceuticals, Ltd., Dearborn, MI, USA) to euthanize pigs. Colon digesta were collected from the distal colon of pigs on d 5 and 11 PI and snap-frozen in liquid nitrogen and stored at −80 °C for untargeted metabolomics and microbiome analysis.

### Untargeted metabolomics analysis

The untargeted metabolomics analysis was performed by the West Coast Metabolomics Center at the University of California, Davis using gas chromatography (Agilent 6890 gas chromatograph controlled using Leco ChromaTOF software version 2.32, Agilent, Santa Clara, CA, USA) coupled with time-of-flight mass spectrometry (GC/TOF-MS) (Leco Pegasus IV time-of-flight mass spectrometer controlled using Leco ChromaTOF software version 2.32, Leco, Joseph, MI, USA). Metabolite extraction was performed following procedures described previously by Fiehn et al. [19]. Briefly, frozen colon digesta samples (approximately 30 μL and 10 mg, respectively) were homogenized using a Retsch ball mill (Retsch, Newtown, PA, USA) for 30 s at 25 times/s. After homogenization, a prechilled (−20 °C) extraction solution (isopropanol/acetonitrile/water at the volume ratio 3:3:2, degassed with liquid nitrogen) was added at a volume of 1 mL/20 mg of sample. Samples were then vortexed and shaken for metabolite extraction. After centrifugation at 12,800 × *g* for 2 min, the supernatant was collected and divided into two equal aliquots and concentrated at room temperature for 4 h in a cold-trap vacuum concentrator (Labconco Centrivap, Kansas City, MO, USA). To separate complex lipids and waxes, the residue was re-suspended in 500 µL of 50% aqueous acetonitrile and centrifuged at 12,800 × *g* for 2 min. The resultant supernatant was collected and concentrated in the vacuum concentrator. Dried sample extracts were derivatized and mixed with internal retention index markers (fatty acid methyl esters with the chain length of C8 to C30). The samples were injected for GC/TOF analysis, and all samples were analyzed in a single batch. Data acquisition by mass spectrometry and mass calibration using FC43 (perfluorotributylamine) before starting analysis sequences. Metabolite identifications were performed based on the two parameters: 1) retention index window ± 2,000 U (around ± 2 s retention time deviation), and 2) mass spectral similarity plus additional confidence criteria as detailed below (Data and statistical analysis). A detailed methodology for data acquisition and metabolite identification is described in a previously published article by Fiehn et al. [19].

### Gut microbiota in distal colon digesta

Bacterial DNA was extracted from distal colon digesta samples using the Quick-DNA Fecal/Soil Microbe Kit (Zymo Research, Irvine, CA, USA), following the manufacturer’s instructions. Extracted bacterial DNA was amplified with PCR, targeting the V4 region of the 16S rRNA gene with primers 515 F (5′-XXXXXXXX*GT*GTGCCAGCMGCCGCGGTAA-3′) with an 8 bp barcode (X) and Illumina adapter (*GT*) and 806 R (5′-GGACTACHVGGGTWTCTAAT-3′) [20]. Amplification included thermocycling conditions of 94 °C for 3 min for denaturation, 35 cycles of 94 °C for 45 s, 50 °C for 1 min, 72 °C for 1.5 min, and 72 °C for 10 min (final elongation). To reduce PCR bias, each sample was amplified in triplicate. Each PCR reaction included 2 μL of template DNA, 0.5 μL (10 μmol/L) of barcoded forward primer, 0.5 μL (10 μmol/L) of reverse primer, 12.5 μL of GoTaq 2× Green Master Mix (Promega, Madison, WI, USA), and 9.5 μL of nuclease-free water. The triplicate PCR products were pooled and subjectively quantified based on the brightness of the bands on a 2% agarose gel with SYBR safe (Invitrogen, Carlsbad, CA, USA). All amplicons were then pooled at equal amounts and further purified using the QIAquick PCR Purification Kit (Qiagen, Hilden, Germany). The purified library was submitted to the UC Davis Genome Center DNA Technologies Core for 250 bp paired-end sequencing on the Illumina MiSeq platform (Illumina, Inc., San Diego, CA, USA).

The software sabre (https://github.com/najoshi/sabre) was used to demultiplex and remove barcodes from raw sequences. Sequences were then imported into Quantitative Insights Into Microbial Ecology 2 (QIIME2; version 2018.6) for downstream filtering and bioinformatics analysis [21, 22]. Plugin q2-dada2 [23] was used for quality control and constructing features. Taxonomic classification was assigned using the feature-classifier plugin trained with the SILVA rRNA database 99% Operational Taxonomic Units (OTU), version 132 [24, 25].

### Data and statistical analysis

The metabolomics data were analyzed using various modules of the web-based platform MetaboAnalyst 5.0 (https://www.metaboanalyst.ca) [26]. Data were filtered to exclude peaks with detection rates lower than 30% and normalized using logarithmic transformation and auto-scaling. Mass univariate analysis was performed using a volcano plot, which combines results from fold change (FC) analysis and *t*-tests. Statistical significance was set at a *P*-value threshold of 0.05 and a fold change threshold of 2.0. Orthogonal partial least squares discriminant analysis (Ortho-PLS-DA) was conducted to further identify discriminative variables (metabolites) among the treatment groups. Pathway analysis and metabolite set enrichment analysis were performed on the identified metabolites that had a variable importance in projection (VIP) score greater than 1. The pathway with a *P*-value less than 0.05, as well as an impact value greater than 0.1, was defined as a significant impact pathway.

Data visualization and statistical analysis for colon microbiota were conducted using R (version 3.6.1). Two alpha diversity indices, Chao1 and Shannon, were calculated using the phyloseq package. Relative abundance was calculated using the phyloseq package and visualized using the ggplot2 package in R. Relative abundance data were aggregated at various taxonomical levels. Shapiro–Wilk normality test and Bartlett test were used to verify normality and constant variance, respectively, in alpha diversity and relative abundance. Shannon index was analyzed using ANOVA with the statistical model, including sample collection days within treatment as fixed effects. Significance in Chao1 index and relative abundance was observed using Kruskal–Wallis rank-sum test followed by a Conover test for multiple pairwise comparisons using the agricolae package. Beta diversity was calculated based on the Bray-Curtis dissimilarity for principal coordinates analysis (PCoA). The homogeneity of multivariate dispersions was tested by the vegan package using the betadisper function before the adonis function was used to calculate PERMANOVA with 999 replicate permutations. Statistical significance and tendency were considered at *P* < 0.05 and 0.05 ≤ *P* < 0.10, respectively.

## Results

### Metabolite profiles in distal colon digesta

**Fig. 1:**
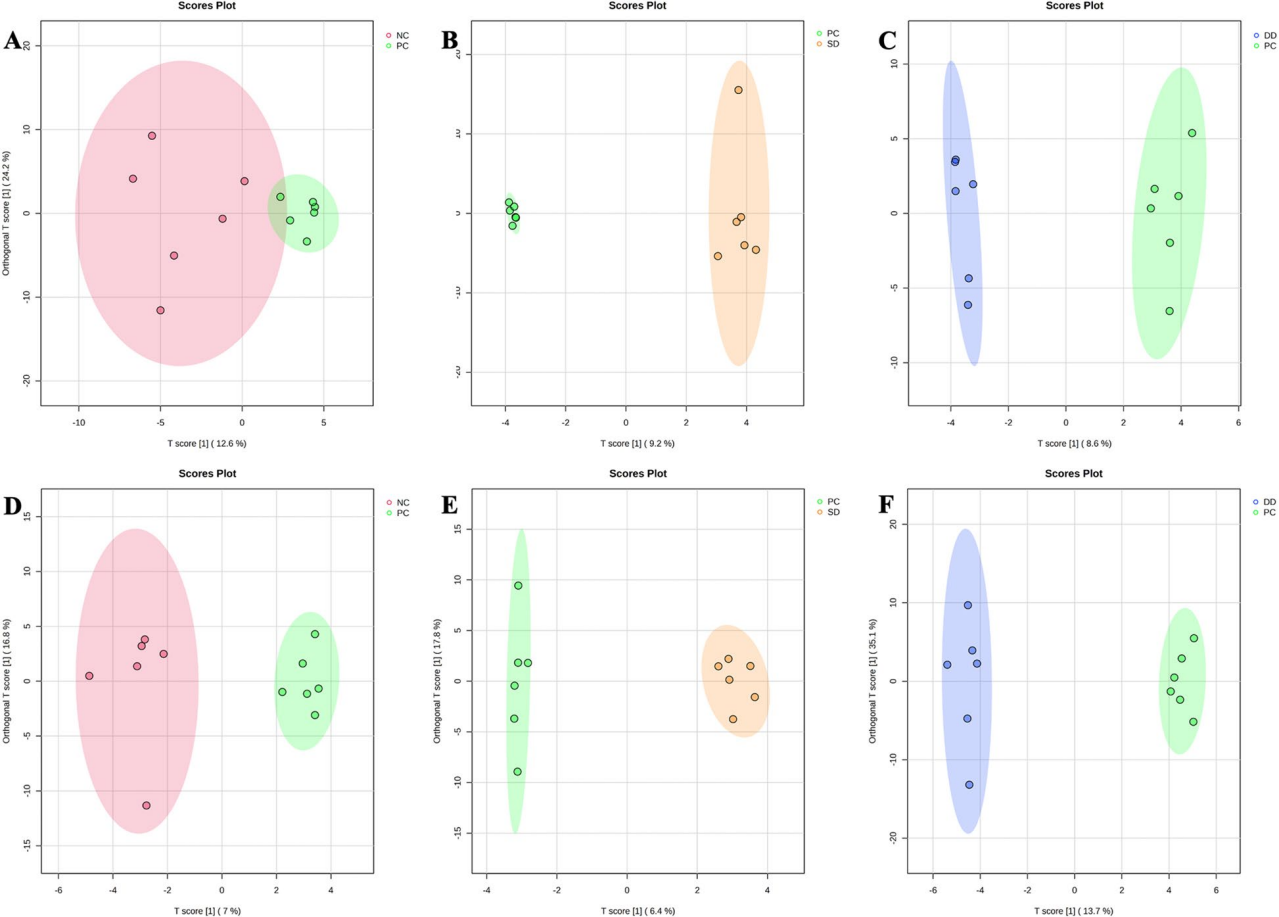
Orthogonal partial least squares discriminant analysis (Ortho-PLS-DA) 2D score plot of the metabolites in distal colon digesta showed a clear separation between the NC vs. PC, PC vs. SD, and PC vs. DD groups on d 5 (**A**–**C**), and d 11 (**D**–**F**) PI, respectively. NC: Negative control; PC: Positive control; SD: Single dose *Bacillus subtilis*; DD: Double dose *Bacillus subtilis*. Shaded areas in different colors represent in 95% confidence interval

A total of 457 (168 identified and 289 unidentified) metabolites were detected in distal colon digesta samples. On d 5 PI, five metabolites (2,6-diaminopimelic acid, ribose, isothreonic acid, 2-aminobutyric acid, and uric acid) were downregulated in the NC group compared with PC (Table 1). Pigs in the PC group had higher levels of 2-aminobutyric acid, proline, 2-hydroxyglutaric acid, spermidine, and pipecolinic acid compared with SD. Similarly, xylulose and ribose were enriched in the PC group relative to DD. Pigs in the SD or DD group (BS; *Bacillus subtilis*) had lower pyruvic acid, 2-aminobutyric acid, and isolinoleic acid compared with PC. On d 11 PI, cellobiose and deoxycholic acid were reduced in the NC group compared with PC, while orotic acid was enriched. Pigs fed BS exhibited reduced 4-hydroxyphenylacetic acid, N-acetylornithine, indole-3-acetate, and dihydro-3-coumaric acid compared with PC. Based on the identified metabolites, Ortho-PLS-DA score plots with 95% confidence ranges (shaded areas) showed a clear separation between the NC vs. PC, PC vs. SD, and PC vs. DD groups throughout the experiment (Fig. 1).

**Table 1:** Distal colon digesta metabolites that differed among the dietary treatment groups

Metabolite	Fold change^1^	VIP^2^	*P*-value
NC^3^ vs. PC^4^, d 5 PI			
2,6-Diaminopimelic acid	0.423	2.12	< 0.05
Ribose	0.445	1.85	< 0.05
Isothreonic acid	0.488	1.71	< 0.05
2-Aminobutyric acid	0.388	1.90	< 0.05
Uric acid	0.435	1.93	< 0.01
PC vs. SD^5^, d 5 PI			
2-Aminobutyric acid	3.787	2.50	< 0.01
Proline	2.371	2.20	< 0.05
2-Hydroxyglutaric acid	2.381	2.03	< 0.05
Spermidine	2.069	2.02	< 0.05
Pipecolinic acid	4.586	1.98	< 0.05
PC vs. DD^6^, d 5 PI			
Xylulose	2.045	2.75	< 0.01
Ribose	2.086	2.66	< 0.01
PC vs. BS^7^, d 5 PI			
Pyruvic acid	2.020	2.25	< 0.01
2-Aminobutyric acid	2.105	1.98	< 0.05
Isolinoleic acid	3.077	2.13	< 0.05
NC vs. PC, d 11 PI			
Cellobiose	0.456	2.16	< 0.05
Deoxycholic acid	0.403	2.12	< 0.05
Orotic acid	3.348	2.59	< 0.05
PC vs. BS, d 11 PI			
4-Hydroxyphenylacetic acid	7.284	2.12	< 0.05
N-acetylornithine	5.837	2.04	< 0.05
Indole-3-acetate	2.686	2.22	< 0.05
Dihydro-3-coumaric acid	7.185	1.72	< 0.05

^1^Fold change values less than one indicate that the differential metabolites were reduced in the NC compared to PC; PC compared to SD; PC compared to DD; or PC compared to BS, respectively

^2^*VIP* Variable importance in the projection

^3^*NC* Negative control

^4^*PC* Positive control

^5^*SD* Single dose *Bacillus subtilis*

^6^*DD* Double dose *Bacillus subtilis*

^7^*BS* *Bacillus subtilis* (SD and DD)

**Fig. 2:**
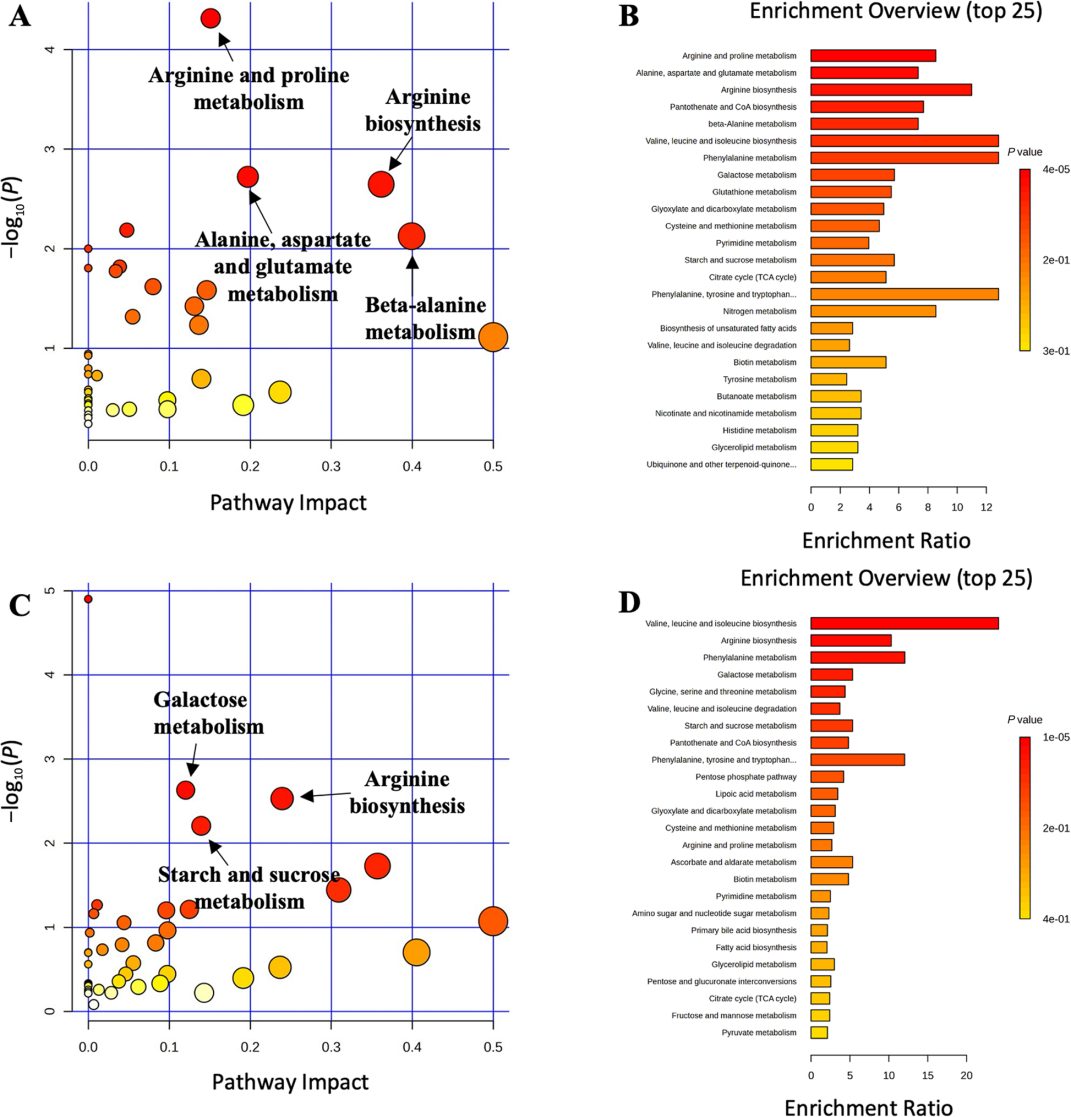
Significantly changed pathways in distal colon digesta between the positive control (PC) and single dose *Bacillus subtilis* (SD) groups (**A**) and PC and double dose *Bacillus subtilis* (DD) groups (**C**) on d 5 post-inoculation. The *x*-axis represents the pathway impact values and the *y*-axis represents the −log_10_(*P*) values from the pathway enrichment analysis. Metabolite set enrichment analysis shows the metabolic pathways were enriched in PC compared to SD, and PC compared to DD on d 5 post-inoculation, respectively (**B** and **D**). Both pathway analysis and metabolite set enrichment analysis were performed using identified metabolites with VIP > 1

**Fig. 3:**
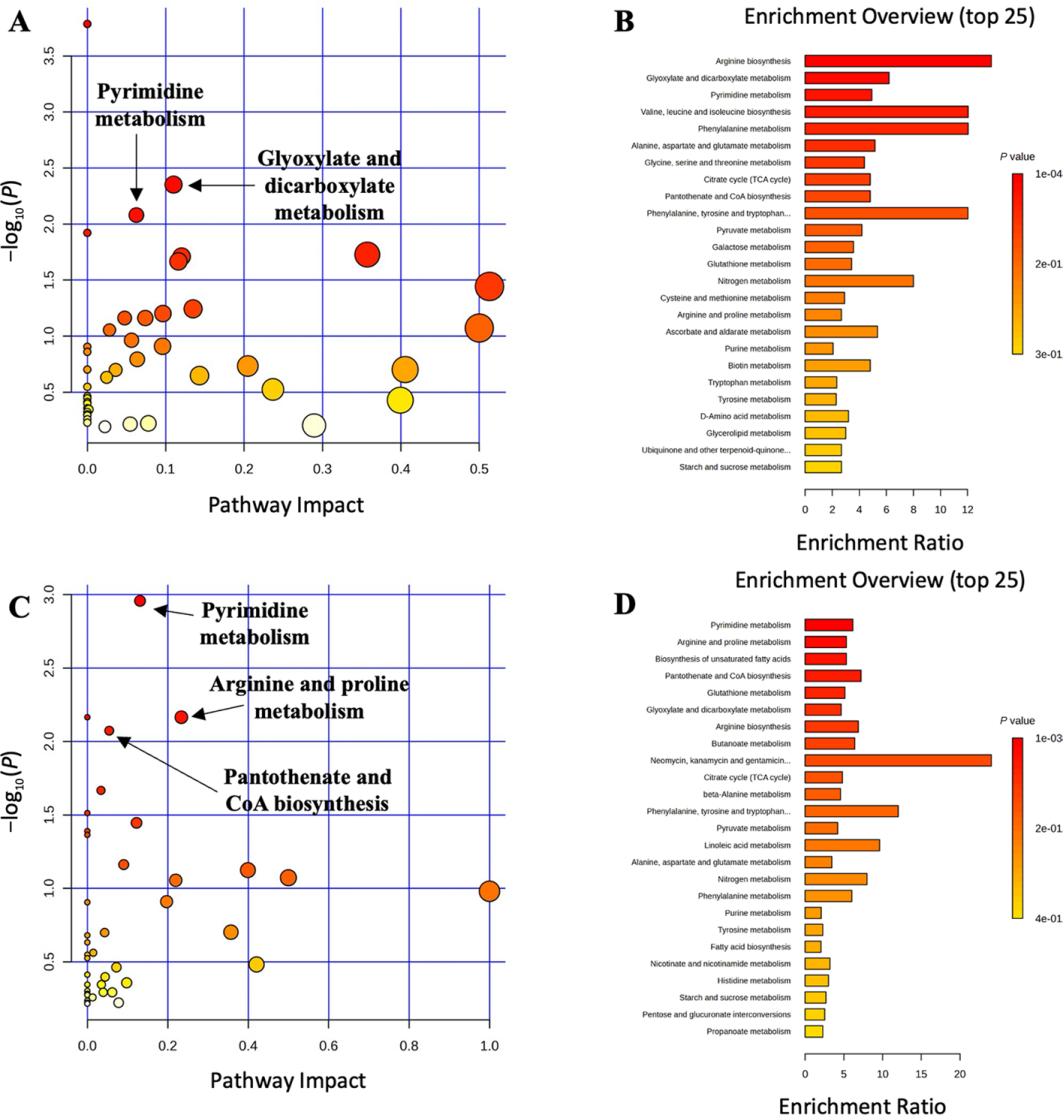
Significantly changed pathways in distal colon digesta between the positive control (PC) and single dose *Bacillus subtilis* (SD) groups (**A**) and PC and double dose *Bacillus subtilis* (DD) groups (**C**) on d 11 post-inoculation. The *x*-axis represents the pathway impact values and the *y*-axis represents the −log_10_(*P*) values from the pathway enrichment analysis. Metabolite set enrichment analysis shows the metabolic pathways were enriched in PC compared to SD, and PC compared to DD on d 11 post-inoculation, respectively (**B** and **D**). Both pathway analysis and metabolite set enrichment analysis were performed using identified metabolites with VIP > 1

Pathway analysis and metabolite set enrichment analysis were performed on metabolites in distal colon digesta with VIP > 1, identifying key metabolic pathways altered by treatment and sampling day. ETEC infection impacted metabolic pathways, including glutathione metabolism, beta-alanine metabolism, arginine and proline metabolism, and pyrimidine metabolism, which were the most affected in the NC vs. PC comparison on d 5 PI (Fig. S1). On d 11 PI, glutathione metabolism, arginine biosynthesis, arginine and proline metabolism, starch and sucrose metabolism, and D-amino acid metabolism were the most altered. On d 5 PI, arginine and proline metabolism, arginine biosynthesis, alanine, aspartate and glutamate metabolism, and beta-alanine metabolism were the most affected pathways in the PC vs. SD comparison, while galactose metabolism, arginine biosynthesis, and starch and sucrose metabolism were impacted in the PC vs. DD comparison (Fig. 2). On d 11 PI, pyrimidine metabolism and glyoxylate and dicarboxylate metabolism were the most altered pathways in the PC vs. SD comparison, whereas pyrimidine metabolism, arginine and proline metabolism, and pantothenate and CoA biosynthesis were most affected in the PC vs. DD comparison (Fig. 3). When the SD and DD groups were combined to assess the overall impact of *Bacillus subtilis* supplementation, phenylalanine metabolism, arginine biosynthesis, and phenylalanine, tyrosine, and tryptophan biosynthesis were the most affected pathways on d 5 PI compared to PC (Fig. S2). On d 11 PI, pyrimidine metabolism, arginine biosynthesis, and biosynthesis of unsaturated fatty acids were significantly altered.

### Microbial profiles in distal colon digesta

**Fig. 4:**
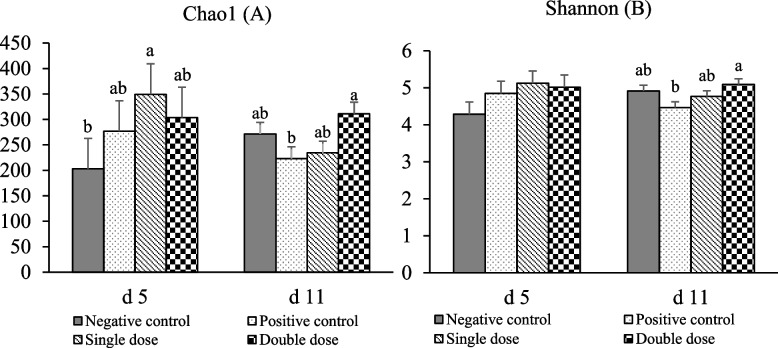
Pig colon digesta alpha diversity, as indicated by Chao1 (**A**) and Shannon (**B**). ^a,b^Means without a common superscript are different (*P* < 0.05). Negative control: Control diet, without enterotoxigenic *Escherichia coli* (ETEC) challenge; Positive control: Control diet, with ETEC challenge; Single dose: Control diet plus 1.28 × 10^9^ CFU/kg *Bacillus subtilis*, with ETEC challenge; Double dose: Control diet plus 2.56 × 10^9^ *Bacillus subtilis*, with ETEC challenge

Pigs in the NC group had less (*P* < 0.05) Chao1 diversity index compared to SD group on d 5 PI, while PC group had less (*P* < 0.05) Chao1 and Shannon diversity index than pigs in the DD group on d 11 PI (Fig. 4). Beta diversity (Adonis analysis based on the Bray-Curtis distance) indicated that days PI was a significant factor associated with compositional distance (*R*^2^ = 0.08, *P* < 0.05; Fig. S3). The interaction effect of treatment and day was also significant (*R*^2^ = 0.18, *P* < 0.05).

**Fig. 5:**
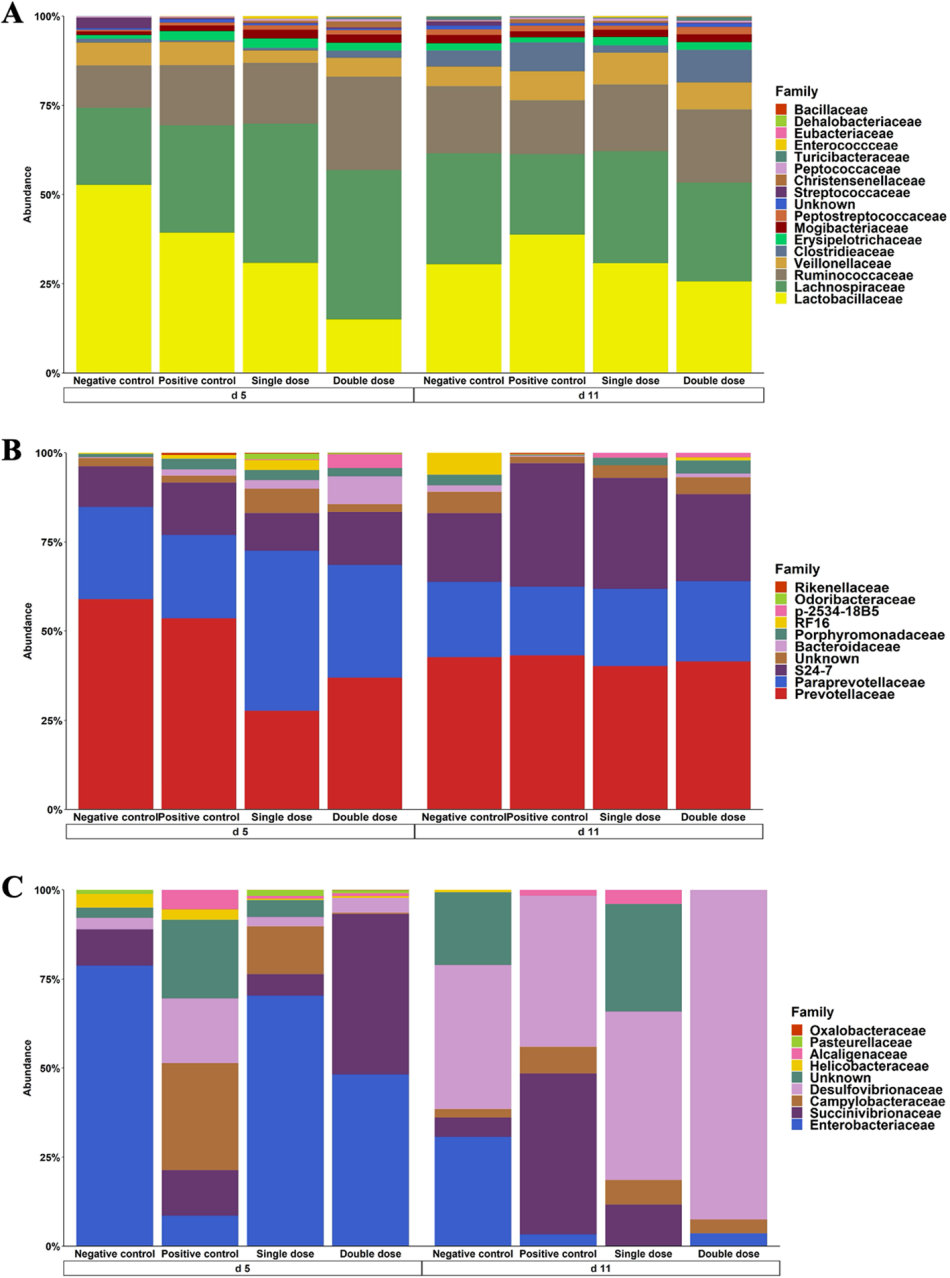
Stacked bar plot showing the relative abundance of Firmicutes (**A**), Bacteroidetes (**B**), and Proteobacteria (**C**) family in colon digesta of enterotoxigenic *Escherichia coli* F18 challenged pigs fed diets supplemented with different doses of *Bacillus subtilis* on d 5 and 11 post-inoculation. Negative control: Control diet, without ETEC challenge; Positive control: Control diet, with ETEC challenge; Single dose: Control diet plus 1.28 × 10^9^ CFU/kg *Bacillus subtilis*, with ETEC challenge; Double dose: Control diet plus 2.56 × 10^9^ *Bacillus subtilis*, with ETEC challenge

The 3 dominant phyla in the distal colon were Firmicutes, Proteobacteria, and Bacteroidetes (Fig. S4). On d 5 PI, Firmicutes abundance was significantly lower (*P* < 0.01) in the SD (69.17%) and DD (62.55%) groups compared to the NC (82.98%) and PC (81.82%). Proteobacteria abundance was significantly higher (*P* < 0.01) in the DD group (14.67%) than in the NC (6.16%) and PC (3.34%). Spirochaetes abundance was also higher (*P* < 0.05) in the DD group (2.56%) compared to the NC (0.45%). No significant differences in relative phyla abundance were observed across treatments on d 11 PI. Within the Firmicutes family on d 5 PI, Lachnospiraceae abundance was significantly higher (*P* < 0.05) in the DD group (46.23%) compared to the NC (21.69%) and PC (29.85%) (Fig. 5A). Lactobacillaceae abundance was significantly (*P* < 0.05) lower in the SD (21.70%) and DD (17.40%) groups compared to the NC (51.74%). Streptococcaceae abundance was significantly lower (*P* < 0.05) in the PC (0.19%), SD (0.27%), and DD (0.54%) groups compared to the NC (3.15%). No significant differences were observed across treatments for these families on d 11 PI. Within the Bacteroidetes family on d 5 PI, Paraprevotellaceae was higher (*P* < 0.05) in the SD group (45.36%) compared with the NC (26.77%), or PC (19.72%) groups (Fig. 5B). Bacteroidaceae exhibited a marked increase (*P* < 0.05) in the DD group (11.21%), whereas the other three groups remained below 2%. By d 11 PI, no significant differences were observed across treatments for these families. Within the Proteobacteria family, pigs in the PC group showed the lowest (*P* < 0.05) abundance of Enterobacteriaceae (4.84%) on d 5 PI compared with the NC group (68.58%), whereas Helicobacteriaceae was highest (*P* < 0.05) in the PC (10.04%) relative to the SD (0.86%) and DD (0.21%) groups (Figs. S4 and S5). Pigs in the DD group exhibited a higher abundance of Desulfovibrionaceae (95.22%) than any other group (*P* < 0.01) on d 11 PI.

## Discussion

Drawing on our previous findings in this follow-up study, we investigated the impact of dietary *Bacillus subtilis* supplementation on the metabolic profiles and microbial ecology of weanling pigs challenged with ETEC. While it is well established that ETEC infection markedly alters host metabolism and gut microbiota, limited research has explored how *Bacillus subtilis* can reprogram these changes under disease-challenged conditions. Our integrated untargeted metabolomics and 16S rRNA gene sequencing analyses revealed that ETEC challenge induced profound alterations in both intestinal metabolite composition and microbial community structure. Notably, *Bacillus subtilis* supplementation led to distinct modifications in metabolic and microbial profiles that suggest an active reprogramming of host–microbe interactions.

### Changes in metabolites in distal colon

ETEC challenge induced a broad spectrum of metabolic disruptions that compromise intestinal homeostasis. Our data showed that ETEC infection modulated glutathione metabolism, a critical antioxidant pathway, suggesting that the pathogen elevates oxidative stress in the intestinal mucosa [27]. The observed dysregulation of glutathione and its precursors aligns with previous studies linking ETEC infections to increased oxidative damage and inflammation in pigs [28–30]. Furthermore, ETEC infection disrupted both beta‐alanine and pyrimidine metabolism, emphasizing extensive metabolic stress in the distal colonic epithelium. Beta‐alanine, a product of pyrimidine catabolism and a key component in pantothenic acid (vitamin B_5_) and CoA biosynthesis, was significantly altered, suggesting downstream effects on energy metabolism and enzyme co-factor availability [31]. Although direct evidence of ETEC affecting beta‐alanine is limited, this disturbance likely reflects the overall metabolic strain and increased nucleotide turnover during infection [32]. Similarly, changes in pyrimidine metabolism indicate elevated cell turnover or enhanced DNA/RNA repair due to mucosal damage. He et al. [5] reported that even after ETEC clearance, nucleotide-related metabolites remained altered compared to pigs not challenged with ETEC, highlighting the lasting impact on gut recovery. In addition, changes in arginine and proline metabolism and arginine biosynthesis are particularly important given that arginine is a precursor for nitric oxide, which is a critical mediator of immune responses [33], while proline is essential for collagen synthesis and tissue repair [34]. Disruptions in carbohydrate metabolism, evident through altered starch and sucrose metabolism, suggest that ETEC infection compromises carbohydrate digestion and energy availability, potentially contributing to the growth setbacks observed in infected piglets [35, 36]. These findings indicate that ETEC-induced dysbiosis favors fermentative microbial populations, thereby further impairing carbohydrate digestion and energy extraction in the gut [37]. Finally, modifications in D-amino acid metabolism imply shifts in both host and microbial processes, as D-amino acids, derived in part from bacterial cell wall turnover, play a role in host–microbe interactions [38]. Although data on ETEC specifically affecting D-amino acids in pigs are limited, it is known that gut dysbiosis can lead to accumulation of microbial D-amino acids. Overall, the metabolic shifts in the distal colon of ETEC-challenged pigs point to a state of oxidative stress, impaired energy and nucleotide balance, amino acid catabolic stress, and altered microbiota function. These findings are consistent with previous reports of ETEC’s multifaceted impact on piglets, including increased oxidative stress, intestinal inflammation, and nutrient malabsorption [4, 5, 39].

Interestingly, dietary *Bacillus subtilis* supplementation induced a distinct metabolic profile in the distal colon that contrasts sharply with the catabolic disruptions caused by ETEC infection. *Bacillus subtilis*-treated pigs under ETEC challenge exhibited significant enrichment in anabolic pathways, particularly in arginine biosynthesis and the urea cycle, thereby restoring nitrogen balance and enhancing arginine availability, which are key for intestinal repair and immune function via nitric oxide production [40]. A previous study also showed that pigs receiving *Bacillus subtilis* had increased activity in arginine biosynthesis and urea cycle pathways in their colon digesta compared to unsupplemented, ETEC-challenged animals [5]. The modification of arginine and proline metabolism appears to counteract the arginine depletion typically observed with ETEC infection, supporting earlier reports that supplemental arginine or glutamine can mitigate infection severity [39, 41]. In this sense, *Bacillus subtilis* may act indirectly, similar to those amino acid supplements, by promoting endogenous arginine-generating pathways or reducing arginine catabolism during infection. Moreover, the impact of *Bacillus subtilis* extended to amino acid metabolism, particularly in the alanine, aspartate, and glutamate pathways, which form a central hub linking glycolysis, the TCA cycle, and nitrogen shuttling [42]. Enhanced activity in these pathways likely supports gluconeogenesis and energy production in enterocytes, thereby improving nitrogen exchange between the host and its microbiota and contributing to better intestinal integrity [43]. In an in vitro study, a *Bacillus subtilis* significantly modulated the alanine, aspartate, and glutamate metabolism in pig intestinal epithelial cells​ [44]. Our in vivo results mirror this trend, suggesting that *Bacillus subtilis* can influence these amino acids in the colonic environment as well. Enhanced metabolism of these amino acids benefits the host in several ways: aspartate serves as a precursor for asparagine, which is important for mucosal health, and for the urea cycle [45]; supporting energy production through glutamate [46]; and facilitating gluconeogenesis and nitrogen exchange via alanine [47]. These improvements are consistent with enhanced intestinal integrity, suggesting that stimulation of these amino acid pathways by *Bacillus subtilis* supports mucosal recovery and overall energy status in ETEC-challenged pigs.

In addition, *Bacillus subtilis* supplementation regulated carbohydrate metabolism by restoring pathways involved in galactose, starch, and sucrose metabolism. In contrast to ETEC-challenged controls, which exhibited malabsorptive fermentation marked by excess luminal sugars and lactic acid accumulation, *Bacillus subtilis*-treated pigs demonstrated improved carbohydrate utilization [48, 49]. This regulation is critical for efficient energy extraction, ensuring that both enterocytes and immune cells receive a steady energy supply during recovery [50]. Such improvements may stem from the *Bacillus subtilis* ability to stabilize the gut environment and contribute digestive enzymes, thereby promoting the production of beneficial short-chain fatty acids instead of lactate [51, 52]. The net effect is a shift in colonic metabolism by *Bacillus subtilis* supplementation toward more efficient energy extraction and less accumulation of disruptive osmolytes, helping to alleviate diarrhea. In addition, *Bacillus subtilis* supplementation was associated with alterations in energy-related pathways, notably pantothenate and CoA biosynthesis, and exhibited distinct changes in pyrimidine metabolism compared to ETEC infection alone. This enhancement suggests an improved capacity for energy metabolism in the colon, potentially through increased production of pantothenic acid by the *Bacillus subtilis* or by the altered microbial community, which may lead to better mitochondrial function and overall energy production. Unlike the damage-driven pyrimidine alterations observed with ETEC infection, the probiotic’s effect on this pathway may reflect improved epithelial cell renewal and more balanced nucleic acid synthesis, thereby supporting tissue repair. Both in vivo and in vitro studies by Sudan et al. [44, 53] demonstrated that *Bacillus subtilis* application in nursery pigs or epithelial cells enriched pathways related to fatty acid oxidation, energy metabolism, and pyrimidine metabolism. This indicates a higher energetic throughput in the gut and supports epithelial recovery by enhancing nucleic acid synthesis while reducing aberrant cell turnover. Our observations of enhanced pantothenate and CoA pathway activity, along with modulation of pyrimidine metabolism, align with these findings and highlight a unique facet of *Bacillus subtilis* action in boosting the host's metabolic co-factors for improved resilience against ETEC. Sudan et al. [53] also reported that *Bacillus subtilis* supplementation in nursery pigs enriched pathways related to fatty acid oxidation, energy metabolism, and pyrimidine metabolism, indicating a higher energetic throughput in the gut and aiding recovery of the epithelial lining by supporting nucleic acid synthesis and reducing the aberrant cell turnover seen in infection. Our observation of altered pantothenate and CoA pathway activity and pyrimidine metabolism aligns with these findings and highlights a unique facet of *Bacillus subtilis* action in enhancing the host's metabolic co-factors for improved resilience against ETEC.

### Changes in microbial community in distal colon

The pig gastrointestinal tract hosts a complex and dynamic microbial ecosystem that plays a critical role in overall health and performance. A balanced microbial community, characterized by high diversity and stability, is essential for efficient nutrient digestion, energy extraction, immune modulation, and maintenance of the intestinal barrier. In healthy pigs, this equilibrium supports robust growth and disease resistance, whereas disruptions in community composition, such as reduced diversity or the overgrowth of pathogenic species, can predispose animals to metabolic inefficiencies, inflammation, and gastrointestinal disorders.

ETEC infection in pigs leads to significant dysbiosis in the distal colon, with marked modifications in key bacterial groups critical for gut function and nutrient metabolism. During the peak infection period (d 5 PI), PC group pigs exhibited an overall reduction in Proteobacteria. However, within this phylum, there was a notable increase in Helicobacteriaceae and a decrease in Enterobacteriaceae compared to healthy controls. Helicobacteriaceae, which are typically mucosa-associated and capable of thriving in microaerophilic environments, may be favored by the inflammatory conditions and altered oxygen tension induced by ETEC, thereby contributing to sustained low-grade inflammation and mucosal disruption [54, 55]. Conversely, the lower abundance of Enterobacteriaceae, despite the fact that this family includes pathogenic *E. coli*, may result from competitive exclusion by other microbial groups as well as the flushing effect of diarrhea and rapid intestinal transit, which reduce their presence in the lumen [56]. Likewise, the relative abundance of Streptococcaceae within the Firmicutes was reduced in ETEC-infected pigs compared to those under normal conditions, further impairing the gut's capacity for carbohydrate fermentation and short-chain fatty acid production [57, 58]. Together, these microbial shifts likely exacerbate impaired nutrient digestion, promote inflammatory responses, and contribute to the clinical manifestations of ETEC infection, such as diarrhea and reduced growth performance, which is consistent with previous findings using same ETEC strain [11, 59, 60].

Dietary *Bacillus subtilis* supplementation in ETEC-challenged pigs has been shown to restore microbial diversity and promote a balanced gut ecosystem. Studies have demonstrated that direct-fed microbials, including *Bacillus subtilis*, help preserve or increase alpha diversity, resulting in a microbiota that more closely resembles that of healthy pigs [61, 62]. In our study, probiotic-treated pigs displayed a richer and more even microbial community post-infection, with a shift in beta diversity indicating a partial normalization of the community structure. A consistent outcome of *Bacillus subtilis* supplementation is the enrichment of beneficial commensal bacteria that are typically depleted during ETEC infection. Notably, *Bacillus subtilis* supplementation in the current study had distinct effects on key bacterial families within the Firmicutes phylum, particularly Lachnospiraceae and Lactobacillaceae. Lachnospiraceae, which produce beneficial short-chain fatty acids such as butyrate, are essential for maintaining gut barrier integrity and immune homeostasis​ [63]. ETEC infection often disrupts this balance by reducing the abundance of such beneficial bacteria [4]; however, *Bacillus subtilis* supplementation helped restore Lachnospiraceae levels, suggesting that the probiotic supports the re-establishment of short-chain fatty acid-producing communities during ETEC challenge. Similar results were reported by Qin et al. [10], where pigs infected with ETEC K88 were supplemented with *Bacillus subtilis* MZ-01, further supporting the role of *Bacillus subtilis* in modulating the gut microbiota to favor beneficial fermentative bacteria. In contrast, the relative abundance of Lactobacillaceae decreased with *Bacillus subtilis* treatment, an unexpected finding given that Lactobacilli are generally regarded as beneficial for pathogen inhibition and intestinal barrier reinforcement through acid production and antimicrobial peptides [64]. This reduction in Lactobacillaceae contrasts with some reports in which *Bacillus subtilis* co-supplementation maintained or increased lactobacilli [65], implying that in our study, *Bacillus subtilis* may competitively inhibit certain *Lactobacillus* populations or alter nutrient availability. Thus, while *Bacillus subtilis* boosted Lachnospiraceae, consistent with its role in stabilizing gut function, it concurrently diminished Lactobacillaceae, highlighting a complex modulation of the microbiota that deviates from the typical probiotic synergy reported elsewhere.

Notably, dietary *Bacillus subtilis* supplementation in current study increased the abundance of Bacteroidaceae, a family integral to degrading complex carbohydrates and proteins in the gut, thereby may enhancing nutrient utilization and overall homeostasis​ [66]. This finding aligns with literature showing that *Bacillus subtilis* supplementation in dogs can help recover Bacteroidetes, such as *Bacteroides *spp., after ETEC-induced dysbiosis, in which pathogens had suppressed these fiber-fermenting commensals​ [67]. However, this trend was not aligned with literature showing that *Bacillus subtilis* lowered Bacteroidaceae in pigs. Jinno et al. [65], indicating that the exact mechanisms governing these changes remain unclear and merit further investigation. Interestingly, *Bacillus subtilis* supplementation also markedly reduced Helicobacteriaceae in the colon digesta. Members of this family, which belong to the Proteobacteria phylum, are associated with gastric ulcers and enteric inflammation; their decline implies a healthier microbial profile [68]. Mechanistically, *Bacillus subtilis* is known to secrete antibacterial compounds, such as the peptide antibiotic amicoumacin A, that directly inhibit Helicobacter species and have been shown to help suppress or eradicate Helicobacter infections [69]. Overall, these results suggest that *Bacillus subtilis* modulates the gut microbiota by promoting beneficial bacteria and reducing potentially harmful pathogens, thereby contributing to improved nutrient metabolism and reduced gut inflammation.

## Conclusions

The present study demonstrates that ETEC infection in weaned pigs induces profound metabolic disruptions and significant dysbiosis in the distal colon, characterized by alterations in key pathways along with marked shifts in microbial community composition. Dietary *Bacillus subtilis* supplementation effectively counteracted these adverse effects by restoring metabolic balance and upregulation of energy-related pathways. Moreover, *Bacillus subtilis* promoted healthier gut microbiota by enriching beneficial bacterial families while reducing potentially harmful taxa. These findings highlight the potential of *Bacillus subtilis* as a functional alternative to antibiotics, offering a multifaceted strategy to mitigate ETEC-induced oxidative stress, inflammation, and nutrient malabsorption, ultimately improving intestinal health and growth performance in post-weaning pigs. Future studies should further explore the mechanistic links between microbial modulation and host metabolism to optimize probiotic strategies for disease prevention and health promotion in swine production.

## Supplementary Information

Additional file 1: Fig. 1. Significantly changed pathways in distal colon digesta between the negative control (NC) and positive control (PC) on d 5 and 11 post-inoculation, respectively (A and C). The *x*-axis represents the pathway impact values and the *y*-axis represents the −log_10_(*P*) values from the pathway enrichment analysis. Metabolite set enrichment analysis shows the metabolic pathways were enriched in NC compared to PC on d 5 and 11 post-inoculation, respectively (B and D). Both pathway analysis and metabolite set enrichment analysis were performed using identified metabolites with VIP > 1.

Additional file 2: Fig. 2. Significantly changed pathways in distal colon digesta between the positive control (PC) and *Bacillus subtilis* (BS) on d 5 and 11 post-inoculation, respectively (A and C). The *x*-axis represents the pathway impact values and the *y*-axis represents the −log_10_(*P*) values from the pathway enrichment analysis. Metabolite set enrichment analysis shows the metabolic pathways were enriched in PC compared to BS on d 5 and 11 post-inoculation, respectively (B and D). Both pathway analysis and metabolite set enrichment analysis were performed using identified metabolites with VIP > 1.

Additional file 3: Fig. 3. Beta diversity of colon digesta of enterotoxigenic *Escherichia coli* (ETEC) F18 challenged pigs fed diets supplemented with different doses of *Bacillus subtilis* on day 5 and 11 post-inoculation. Data were analyzed by principal coordinate analysis (PCoA) based on the Bray-Curtis dissimilarity. Symbols indicate dietary treatments and colors indicate different sampling dates. Negative Control: Control diet, without ETEC challenge; Positive Control: Control diet, with ETEC challenge; Single Dose: Control diet plus 1.28 × 10^9^ CFU/kg *Bacillus subtilis*, with ETEC challenge; Double Dose: Control diet plus 2.56 × 10^9^ *Bacillus subtilis*, with ETEC challenge.

Additional file 4: Fig. 4. Stacked bar plot showing the relative abundance of phylum in colon digesta of enterotoxigenic *Escherichia coli* F18 challenged pigs fed diets supplemented with different doses of *Bacillus subtilis* on d 5 and 11 post-inoculation. Negative control: Control diet, without ETEC challenge; Positive control: Control diet, with ETEC challenge; Single dose: Control diet plus 1.28 × 10^9^ CFU/kg *Bacillus subtilis*, with ETEC challenge; Double dose: Control diet plus 2.56 × 10^9^ *Bacillus subtilis*, with ETEC challenge.

## Data Availability

All data generated or analyzed during this study are available from the corresponding author upon reasonable request.

## Abbreviations

DD: Double dose of *Bacillus subtilis*
ETEC: Enterotoxigenic *Escherichia coli*
FC: Fold change
NC: Negative control
SD: Single dose of *Bacillus subtilis*
OTU: Operational taxonomic units
PC: Positive control
PCoA: Principal coordinates analysis
PI: Post-inoculation
VIP: Variable importance in projection
