# Supplementary figures and images for: Impact of dietary supplementation of Bacillus subtilis on the metabolic profiles and microbial ecology of weanling pigs experimentally infected with a pathogenic Escherichia coli
Source: J Anim Sci Biotechnol. 2025 Dec 6;16:167. doi: 10.1186/s40104-025-01313-7 (PMC12681179; doi:10.1186/s40104-025-01313-7)

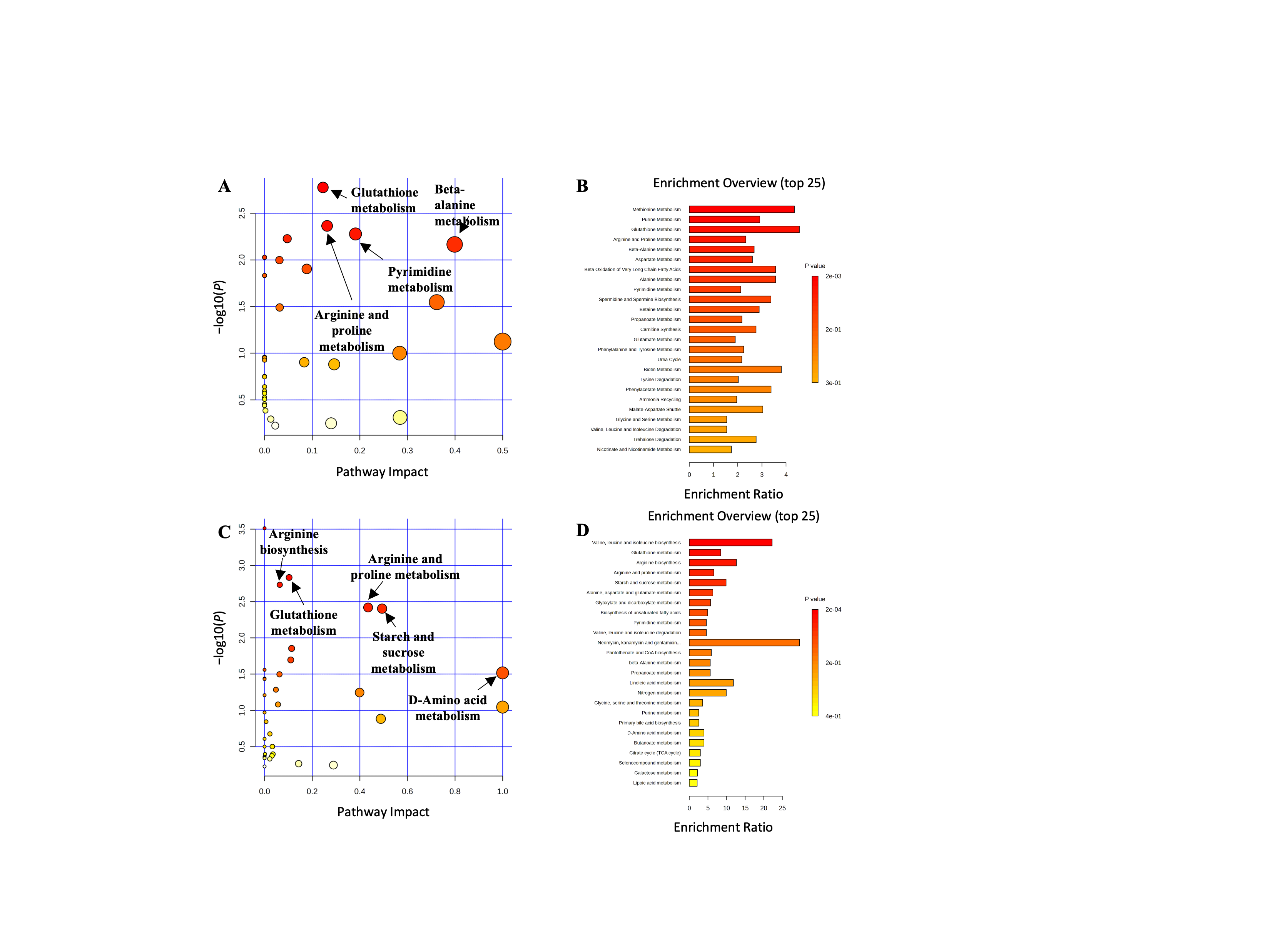

Supplement: Supplementary file 1 — Additional file 1: Fig. 1. Significantly changed pathways in distal colon digesta between the negative control (NC) and positive control (PC) on d 5 and 11 post-inoculation, respectively (A and C). The x-axis represents the pathway impact values and the y-axis represents the −log10(P) values from the pathway enrichment analysis. Metabolite set enrichment analysis shows the metabolic pathways were enriched in NC compared to PC on d 5 and 11 post-inoculation, respectively (B and D). Both pathway analysis and metabolite set enrichment analysis were performed using identified metabolites with VIP > 1. [file 40104_2025_1313_MOESM1_ESM.tiff]

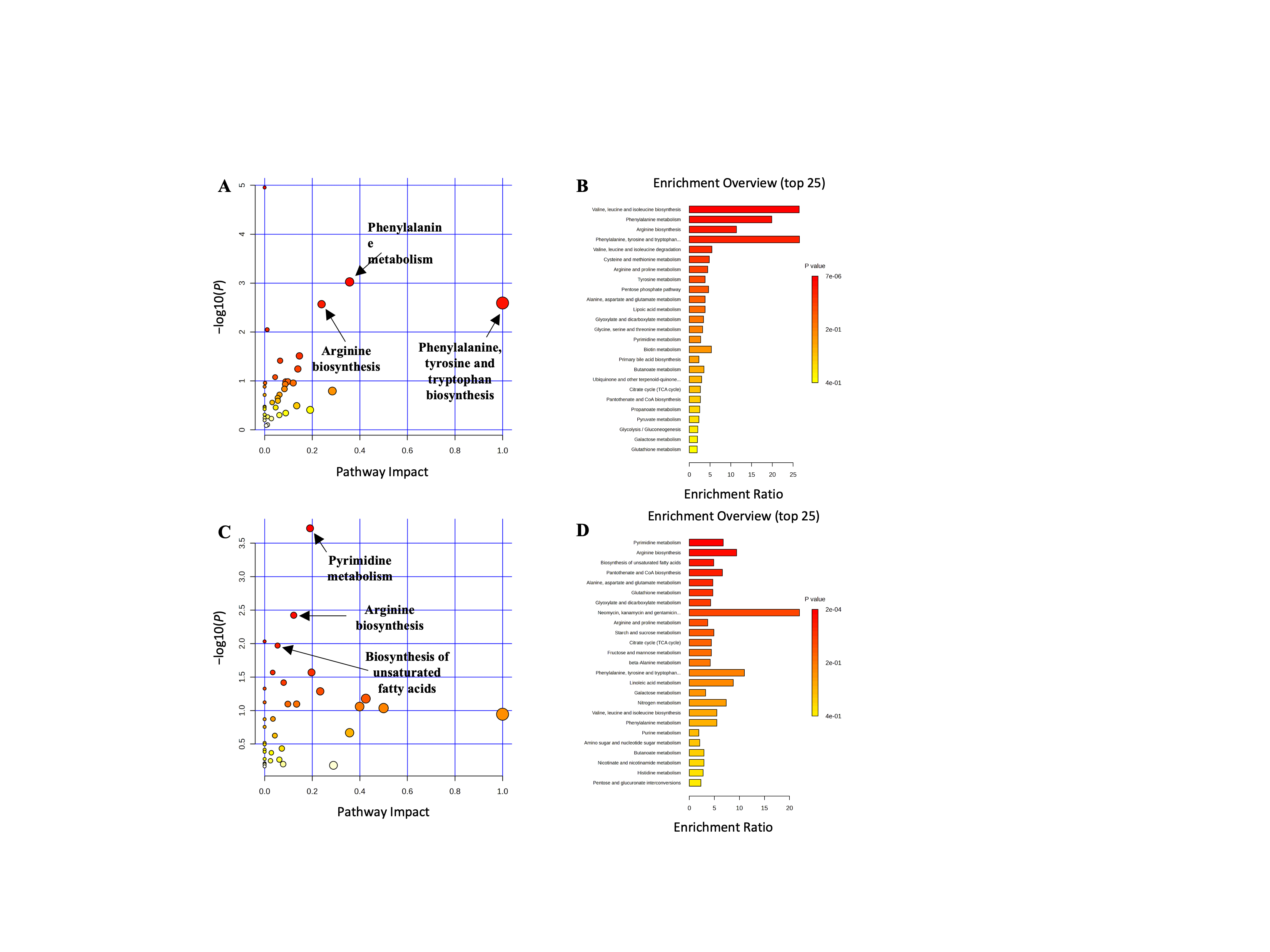

Supplement: Supplementary file 2 — Additional file 2: Fig. 2. Significantly changed pathways in distal colon digesta between the positive control (PC) and Bacillus subtilis (BS) on d 5 and 11 post-inoculation, respectively (A and C). The x-axis represents the pathway impact values and the y-axis represents the −log10(P) values from the pathway enrichment analysis. Metabolite set enrichment analysis shows the metabolic pathways were enriched in PC compared to BS on d 5 and 11 post-inoculation, respectively (B and D). Both pathway analysis and metabolite set enrichment analysis were performed using identified metabolites with VIP > 1. [file 40104_2025_1313_MOESM2_ESM.tiff]

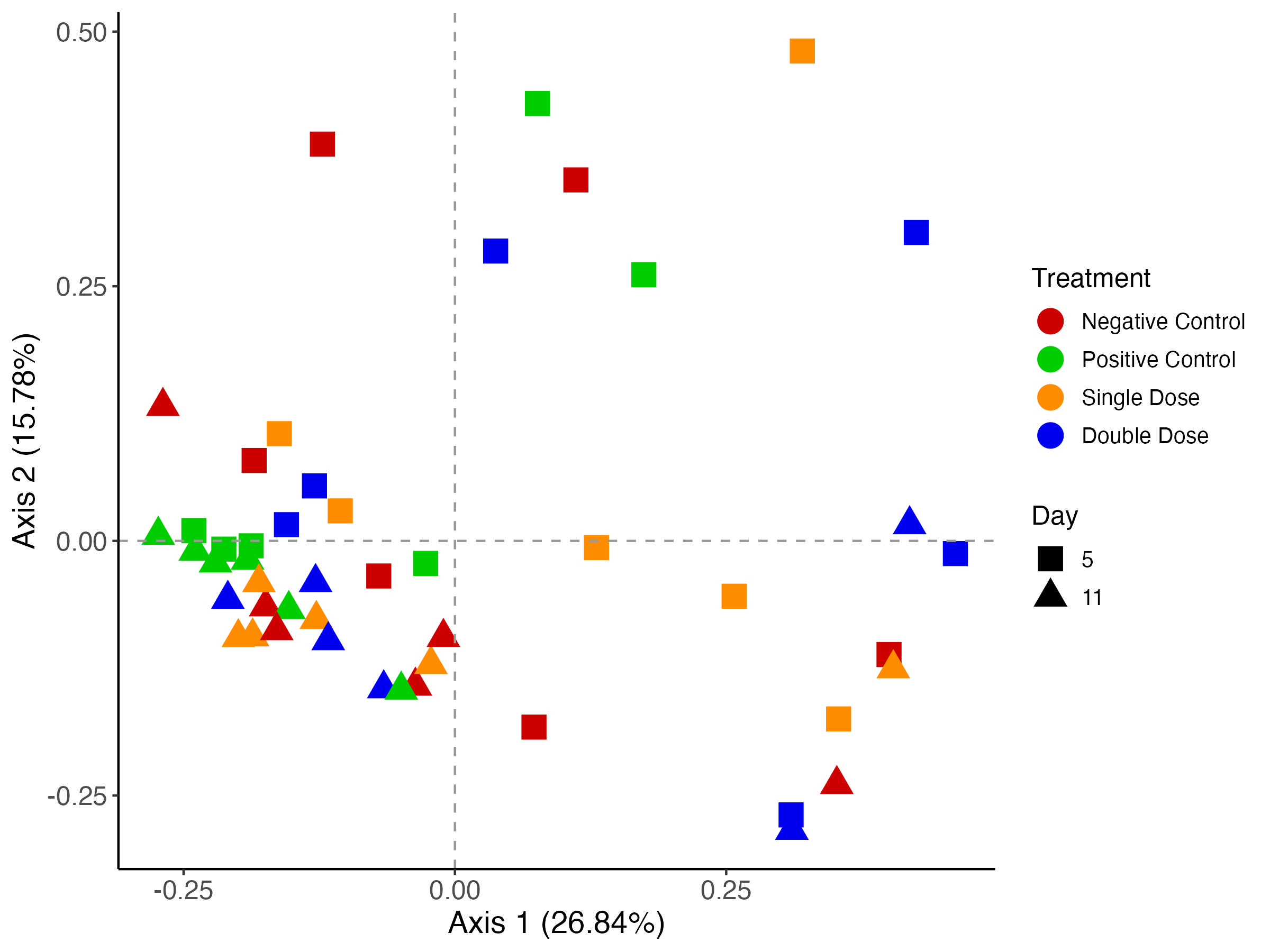

Supplement: Supplementary file 3 — Additional file 3: Fig. 3. Beta diversity of colon digesta of enterotoxigenic Escherichia coli (ETEC) F18 challenged pigs fed diets supplemented with different doses of Bacillus subtilis on day 5 and 11 post-inoculation. Data were analyzed by principal coordinate analysis (PCoA) based on the Bray-Curtis dissimilarity. Symbols indicate dietary treatments and colors indicate different sampling dates. Negative Control: Control diet, without ETEC challenge; Positive Control: Control diet, with ETEC challenge; Single Dose: Control diet plus 1.28 × 10^9^ CFU/kg Bacillus subtilis, with ETEC challenge; Double Dose: Control diet plus 2.56 × 10^9^ Bacillus subtilis, with ETEC challenge. [file 40104_2025_1313_MOESM3_ESM.tiff]

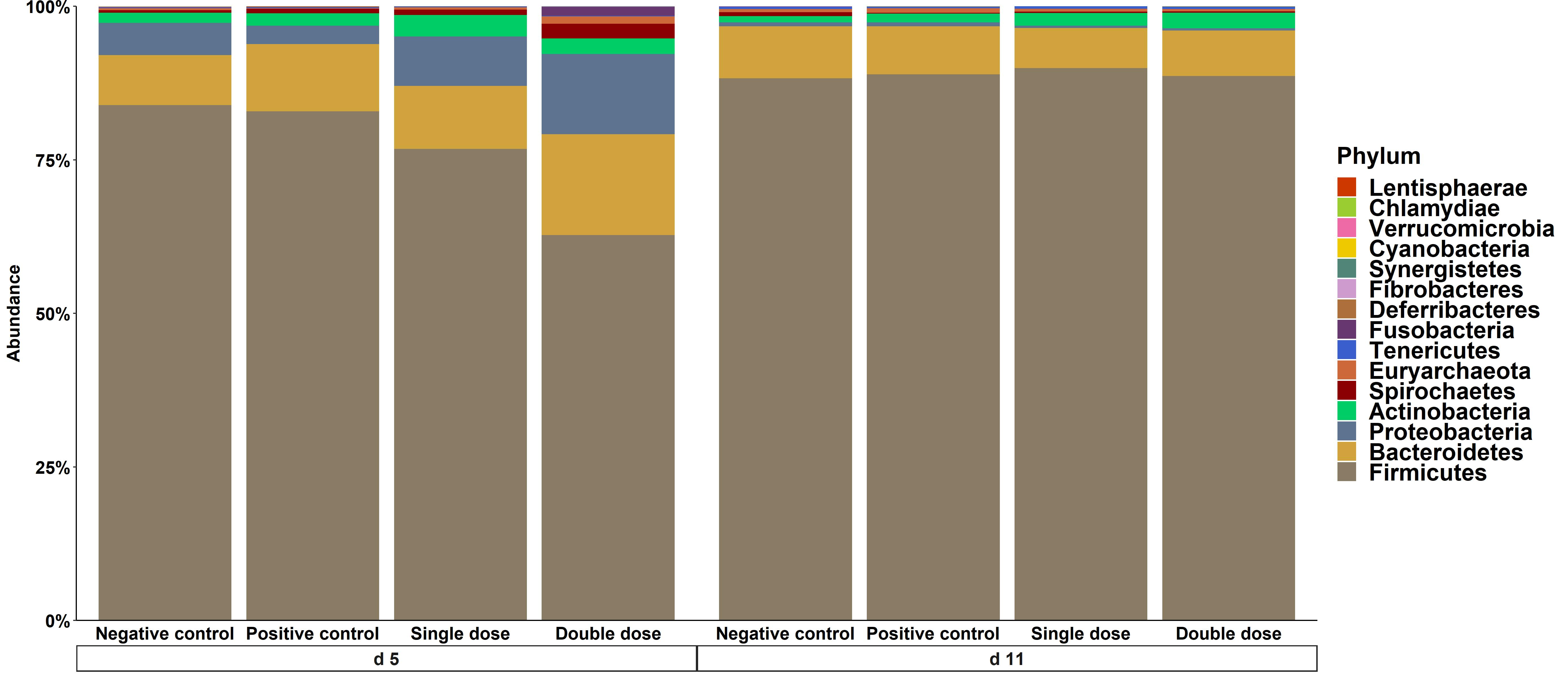

Supplement: Supplementary file 4 — Additional file 4: Fig. 4. Stacked bar plot showing the relative abundance of phylum in colon digesta of enterotoxigenic Escherichia coli F18 challenged pigs fed diets supplemented with different doses of Bacillus subtilis on d 5 and 11 post-inoculation. Negative control: Control diet, without ETEC challenge; Positive control: Control diet, with ETEC challenge; Single dose: Control diet plus 1.28 × 10^9^ CFU/kg Bacillus subtilis, with ETEC challenge; Double dose: Control diet plus 2.56 × 10^9^ Bacillus subtilis, with ETEC challenge. [file 40104_2025_1313_MOESM4_ESM.tiff]
